# Correlation between changes of pelvic bone marrow fat content and hematological toxicity in concurrent chemoradiotherapy for cervical cancer

**DOI:** 10.1186/s13014-022-02029-y

**Published:** 2022-04-07

**Authors:** Cong Wang, Xiaohang Qin, Guanzhong Gong, Lizhen Wang, Ya Su, Yong Yin

**Affiliations:** 1 Department of Fourth Ward of Gynecologic Oncology, Shandong Cancer Hospital and Institute, Shandong First Medical University and Shandong Academy of Medical Sciences, Jinan, China; 2 Department of Graduate, Shandong First Medical University and Shandong Academy of Medical Sciences, Jinan, China; 3 Department of Radiation Physics, Shandong Cancer Hospital and Institute, Shandong First Medical University and Shandong Academy of Medical Sciences, Ji Yan Road No.440, Jinan, 250117 Shandong China

**Keywords:** Bone marrow sparing, IDEAL IQ, Cervical cancer, Chemoradiotherapy

## Abstract

**Objectives:**

To quantify the pelvic bone marrow (PBM) fat content changes receiving different radiation doses of concurrent chemoradiotherapy for cervical cancer and to determine association with peripheral blood cell counts.

**Methods:**

The data of 54 patients were prospectively collected. Patients underwent MRI iterative decomposition of water and fat with echo asymmetrical and least squares estimation (IDEAL IQ) scanning at RT-Pre, RT mid-point, RT end, and six months. The changes in proton density fat fraction (PDFF%) at 5–10 Gy, 10–15 Gy, 15–20 Gy, 20–30 Gy, 30–40 Gy, 40–50 Gy, and > 50 Gy doses were analyzed. Spearman’s rank correlations were performed between peripheral blood cell counts versus the differences in PDFF% at different dose gradients before and after treatment.

**Results:**

The lymphocytes (ALC) nadirs appeared at the midpoint of radiotherapy, which was only 27.6% of RT-Pre; the white blood cells (WBC), neutrophils (ANC), and platelets (PLT) nadirs appeared at the end of radiotherapy which was 52.4%, 65.1%, and 69.3% of RT-Pre, respectively. At RT mid-point and RT-end, PDFF% increased by 46.8% and 58.5%, respectively. Six months after radiotherapy, PDFF% decreased by 4.71% under 5–30 Gy compared to RT-end, while it still increased by 55.95% compared to RT-Pre. There was a significant positive correlation between PDFF% and ANC nadirs at 5–10 Gy (r = 0.62, *P* = 0.006), and correlation was observed between PDFF% and ALC nadirs at 5–10 Gy (r = 0.554, *P* = 0.017).

**Conclusion:**

MRI IDEAL IQ imaging is a non-invasive approach to evaluate and track the changes of PBM fat content with concurrent chemoradiotherapy for cervical cancer. The limitation of low-dose bone marrow irradiation volume in cervical cancer concurrent chemoradiotherapy should be paid more attention to.

## Introduction

Concurrent chemoradiation therapy can improve the overall survival rate and local control rate of patients with cervical cancer, with the 3-year disease-free survival rate possibly reaching 40% (stage IVA)—75% (stage IIB) [1, 2]. Although chemoradiotherapy improves the prognosis of various tumors, the incidence of ≥ 3 hematological toxicity (HT) is approximately 87%. The process of radiotherapy and chemotherapy can be delayed or interrupted because of severe bone marrow suppression [3, 4]. Bone marrow, a heterogeneous mixed tissue, can be divided into red and yellow bone marrow with hematopoietic activity and high-fat content, respectively. Radiation and chemotherapeutic drugs can induce red bone marrow to differentiate into fat cells, leading to red and yellow bone marrow transformation, increasing the bone marrow fat content and inhibiting hematopoietic function [5]. The primary distribution locations of active bone marrow are the pelvis, lumbar spine, and thoracic spine. Moreover, the active bone marrow of the pelvic and lumbar spine is located in the radiation fields of cervical cancer radiotherapy. Studies have demonstrated that the dose limitation of active bone marrow as an organ at risk can effectively reduce the incidence of blood toxicity [6–8]. An international multi-center phase II clinical study confirmed that positron emission tomography (PET/CT)-guided intensity modulated radiotherapy (IMRT) can reduce the incidence of neutropenia above grade 3 from 27 to 8%, while the average bone marrow dose limit was 26.4 Gy [9].

The important strategies to reduce radiation toxicity and improve tolerance are advanced imaging and radiotherapy technologies. The iterative decomposition of water and fat with echo asymmetrical and least squares estimation (IDEAL IQ) imaging of MRI is a fat quantitative three-dimensional scanning technology that can achieve the aim of separating water and fat and assessing bone marrow fat content accurately. Previous studies have shown that fat quantitative MRI is highly sensitive to the bone marrow changes before and after radiotherapy and chemotherapy. Liang et al. used FatFrac imaging coupled with PET/CT to evaluate functional BM that can reduce the risk of HT [10].

The present study used the MRI IDEAL IQ sequence and quantitatively determined the changes in bone marrow fat content in patients with cervical cancer during concurrent chemoradiotherapy. The associations between peripheral blood cell counts and fat content changes were also evaluated to provide a basis for individualized bone marrow sparing.

## Materials and methods

### Patient population and characteristics

A total of 54 patients diagnosed with the International Federation of Gynecology and Obstetrics (FIGO) Stage IB to IVB cervical cancer at Shandong cancer hospital from Jan 2019 to Aug 2020 were prospectively selected for the present study. The patients’ information is shown in Table 1. All patients underwent IMRT or volumetric modulated arc therapy (VMAT) external beam radiotherapy and concurrent cisplatin chemotherapy.

**Table 1:** Patient characteristics

	n (%)
	Total n = 54
*Age*	
Median (range)	51 (20–65)
*Pathology, n (%)*	
SCC	44 (79%)
CAC	10 (21%)
*FIGO stage, n (%)*	
I	18 (33%)
II	14 (26%)
III	21 (39%)
IV	1 (2%)
*Radiotherapy days*	
Median (range)	36 days (31–45 days)
*GTV prescription dose*	
Median (range)	50 Gy (40–65 Gy)

SCC–cervical squamous cell carcinoma

CAC–cervical adenocarcinoma; GTV-gross tumor volume

FIGO–international federation of gynecology and obstetrics

### CT simulation

A Philips 16-slice Brilliance big-bore computed tomography scanner (Philips Medical Systems, Amsterdam, Netherlands) with 3 mm slice thickness and 3 mm slice gap was used for imaging. The scans were made from the upper border of the T2 vertebra to the middle of the femur. Patients were immobilized with thermoplastic mold in a supine position or immobilized with an abdominal pelvic fixator in a prone position.

### MR simulation

All MRI acquisitions were performed on a 3.0-T MRI system (Discovery MR 750, GE Medical System) with the same position and fixed device as CT scans. All patients underwent T1WI, T2WI, and IDEAL IQ serial scans (detailed MR sequences are listed in Table 2). Patients underwent MRI with the same sequence at RT-Pre (before the treatment), RT mid-point, RT end, and six months after treatment.

**Table 2:** Imaging parameters for MRI protocol

Parameters	TR (ms)	TE (ms)	Field of view (cm)	Acquisition matrix (phase × frequency)	Slice thickness (mm)	NEX	Bandwidth	Acquisition time
T1WI	6.5	2.7	36	240 × 300	3.0	1.0	83.333	41 (s)
T2WI	11,593	96	36	352 × 352	3.0	3.0	83.333	7.21 (min)
IDEAL IQ	7.6	Min full	40	180 × 180	3.0	2.0	83.33	2.19 (min)

### Radiation contouring and planning

The CT and MR images were imported into Eclipse15.5 (Varian, USA) planning system for targets and the organs at risk (OARs) contouring. The clinical target volume (CTV) included the gross tumor volume (GTV), uterus, cervix, parametrium, the upper third of the vagina, and locoregional lymph nodes, which included common, internal and external iliac, obturator, and presacral lymph nodes. The corresponding planning target volume (PTV) was generated with 5 mm margins from the CTV. OARs including bladder, rectum, small intestine, spinal cord, delineated the marrow cavity from the lower edge of the L4 vertebral to the end of the femoral heads as pelvic bone marrow. The IMRT or VMAT plan used 6 MV photon beams, and the standard for the acceptance of the plan was that at least 95% volume of the PTV received 100% of the prescription dose. To reduce the impact of tumor shrinkage on OARs, CT and MR simulation were performed again after GTV received 25–36 Gy, and the second treatment plan was designed. To the PTV, all patients received IMRT or VMAT at a dose of 45.0–50.4 Gy in 25–28 daily fractions. Dose-volume constraints for OARs based on the protocol recommended by the EMBRACE II study [11].

### BM fat content calculation

According to the actual radiation dose of each patient, dose maps were generated in the Eclipse treatment planning system. The dose maps, CT and MR scan images, and RT-plans were transmitted into the MIM Maestro (version 6.8.2, USA). The first and second RT plans were rigidly registered to obtain the superimposed RT dose in the MIM Maestro. The dose gradients of 5–10 Gy, 10–15 Gy, 15–20 Gy, 20–30 Gy, 30–40 Gy, 40–50 Gy, and > 50 Gy were obtained.After the dose superimposition, IDEAL IQ FatFrac images were rigidly registered with the CT image to obtain the FatFrac image with dose distribution.

The IDEAL IQ FatFrac images were analyzed using a GE workstation (AW4.7; GE Healthcare). A region of interest (ROI) was ascribed in the iliac bone (excluding cortical edges or blood vessels) under different dose gradients, and the values of bone marrow proton density fat fraction (PDFF) in each ROI were determined. The PDFF value was the ratio of the number of protons in fat to the total number of protons in water and fat within a given ROI. It was expressed as percentages (PDFF%) in the range of 0% to 100%. Each ROI was placed at different positions of the same dose gradient, measured three times and take the average was considered to eliminate measurement errors. The average PDFF% represents the PDFF% under the dose gradient.

### Peripheral blood cells analysis

Blood cell analysis was carried out weekly for all patients during radiotherapy. Absolute counts of white blood cells (WBC), neutrophils (ANC), lymphocytes (ALC), platelets (PLT), and hemoglobin (HGB) were prospectively collected at RT-Pre, at RT mid-point, RT-end, and one month.

### Statistical analysis

The change rate of PDFF% value at RT-end (PDFF%^post^) compared with PDFF% value at RT-Pre (PDFF%^pre^): PDFF% change rate = (PDFF% ^post^ -PDFF%^pre^)/PDFF%^pre^ was calculated. Spearman’s rank correlations were performed by the nadir of peripheral blood cell counts versus bone marrow dose-volume parameters and the changes in PDFF% at different dose gradients. All statistical tests were considered to be statistically significant at *P* < 0.05. The data were analyzed in IBM SPSS Version25 (IBM SPSS Inc, Chicago, IL).

## Results

### Changes in peripheral blood count during treatment

**Fig. 1:**
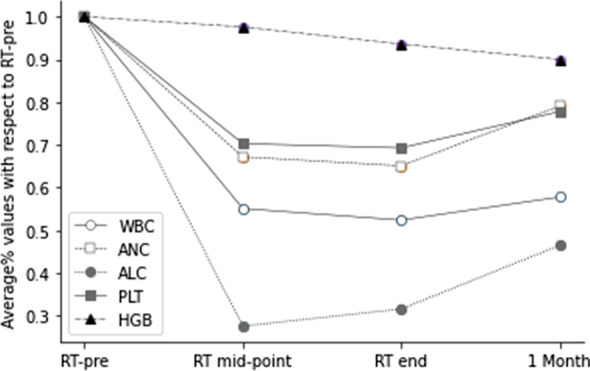
Overall trend of median white blood cell (WBC), neutrophil (ANC), lymphocyte (ALC), platelet (PLT), and hemoglobin (HGB) values with respect to RT-Pre at different time intervals

The values of WBC, ANC, ALC, PLT, and HGB declined significantly during treatment. ALC reached its nadir at RT mid-point, which was only 27.6% of RT-Pre. The WBC, ANC, and PLT nadirs were observed at the end of radiotherapy, and they decreased by 47.6%, 34.9%, and 30.7%, respectively, compared with those at RT-Pre. At the one month after radiotherapy, WBC, ANC, ALC, and PLT increased by 10.3%, 21.5%, 47.2%, and 12.3%, respectively (Fig. 1). The peripheral blood cell counts during the treatment are shown in Table 3.

**Table 3:** Peripheral blood cell counts over the course of treatment

	WBC (× 10^9/L)	ANC (10^9/L)	ALC (× 10^9/L)	PLT (× 10^9/L)	HGB (g/L)
RT-pre	5.99 ± 1.48	4.02 ± 1.34	1.41 ± 0.46	247 ± 72.69	117 ± 13.32
RT-mid	3.20 ± 1.31	2.57 ± 1.54	0.37 ± 0.17	165 ± 54.28	113 ± 12.65
RT-end	2.98 ± 0.89	2.37 ± 1.0	0.43 ± 0.3	161 ± 57.82	108 ± 13.34
1 Mon	3.38 ± 1.16	2.83 ± 1.96	0.6 ± 0.33	177 ± 71.21	103 ± 15.94

### Correlation between PBM dose volume parameters versus peripheral blood cell count

The correlation analysis between dose-volume parameters and peripheral blood cell count (shown in Table 4) indicated that pelvic bone marrow V40 had a negative correlation with the WBC and HGB nadirs (r = –0.364, –0.357, *P* < 0.01). No correlation was seen under other dose gradients. The average volume% of PBM is shown in Table 4.

**Table 4:** Spearman’s rank correlations for the dose-volume parameters versus peripheral blood cell counts

	Volume% (x ± S)	WBC nadirs		ANC nadirs		ALC nadirs		PLT nadirs		HGB nadirs	
		r	*P*	r	*P*	r	*P*	r	*P*	r	*P*
V5	99.52%	− 0.061	.662	− 0.096	.491	0	1	− 0.174	.209	− 0.09	.516
V10	98.05%	0	.999	− 0.046	.74	− 0.105	.448	0.162	.243	0.155	.263
V15	90.86%	− 0.002	.988	0.126	.364	− 0.051	.717	− 0.085	.541	− 0.036	.798
V20	74.08%	− 0.063	.651	0.103	.457	− 0.122	.378	− 0.258	.06	− 0.114	.41
V30	39.67%	− 0.197	.154	− 0.011	.939	− 0.209	.129	− 0.246	.073	− 0.264	.054
V40	16.32%	− 0.364	.007*	− 0.203	.145	− 0.186	.181	0	.999	− 0.357	.009*
V50	5.93%	− 0.05	.756	0.061	.703	0.08	.615	0.035	.824	− 0.226	.151

^*^Statistically significant

### Correlation between changes of PDFF% versus radiation dose between pre-and post-RT

**Fig.2:**
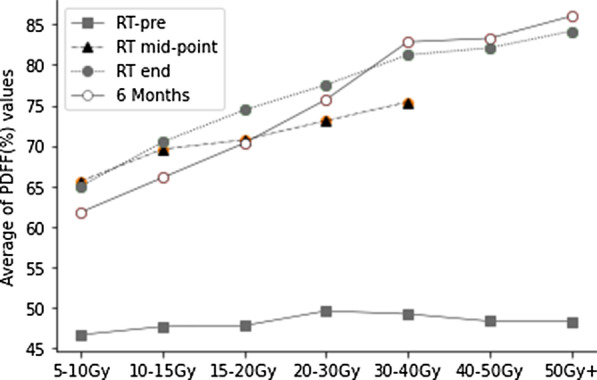
Overall trend of an average of PDFF (%) values with respect to RT-Pre at different dose gradients

The PBM dose-PDFF% profiles at RT-pre, RT mid-point, RT-end, and six months are shown in Fig. 2. The PDFF% values of PBM at RT-Pre were equal and at a low level, with an average of 48.51%. PDFF% increased significantly under all dose gradients during treatment, and PDFF% increased with the dose increase. A correlation was seen between dose accumulate and PDFF% changes (r = 0.411, *P* < 0.01).

At RT mid-point and RT-end, the largest increase in PDFF% was 53.2% and 74.2%, respectively. The average PDFF% was increased by 47% and 58.5%, respectively, compared to RT-Pre at each dose gradient. After six months of radiotherapy, PDFF% values at 5–30 Gy dose gradients were lower than that at RT-end and increased further at 30 Gy + dose gradients. The average of PDFF% increased by 55.95% compared with RT-Pre. PDFF% values over the course of treatment at different dose gradients are shown in Table 5.

**Table 5:** PDFF% values

	5–10 Gy	10–15 Gy	15–20 Gy	20–30 Gy	30–40 Gy	40–50 Gy	> 50 Gy
RT-Pre	46.66 ± 10.3	47.67 ± 10	47.79 ± 11	49.6 ± 11.27	49.23 ± 11.33	48.34 ± 9.53	48.3 ± 10.65
RT mid	65.63 ± 10.98	69.58 ± 9.34	70.73 ± 8.31	73.09 ± 8.55	75.4 ± 8.96	–	–
RT end	65.0 ± 11.44	70.45 ± 10.26	74.42 ± 9.58	77.52 ± 7.74	81.22 ± 6.35	82.08 ± 6.08	84.15 ± 5.1
6 Months	61.79 ± 9.13	66.06 ± 8.69	70.32 ± 10.03	75.7 ± 9.35	82.82 ± 7.74	83.27 ± 6.78	86.0 ± 8.11

### Correlations between the changes of PDFF (%) versus peripheral blood cell counts

We observed correlations between the changes in PDFF (%) versus peripheral blood cell counts nadirs at different dose gradients (Table 6). Significant positive correlations were noted between PDFF (%) and ANC nadirs at 5–10 Gy (r = 0.62, *P* = 0.006). This indicated that increasing fat fraction correlates with decreasing WBC counts throughout treatment. Similarly, correlations between PDFF (%) and ALC nadirs were observed at 5–40 Gy, and significant positive correlations were observed at 5–10 Gy. There are no significant correlations between PDFF (%) and WBC, PLT, and HGB nadirs.

**Table 6:** Spearman’s rank correlations for the difference in PDFF (%) versus peripheral blood cell counts

	WBC nadirs		ANC nadirs		ALC nadirs		PLT nadirs		HGB nadirs	
	r	*P*	r	*P*	r	*P*	r	*P*	r	*P*
5–10 Gy	0.463	0.053	0.620	0.006*	0.554	0.017*	0.309	0.212	0.105	0.677
10–15 Gy	0.208	0.199	0.301	0.06	0.386	0.014*	0.217	0.179	0.025	0.88
15–20 Gy	0.168	0.28	0.188	0.227	0.387	0.01*	0.232	0.134	0.165	0.289
20–30 Gy	0.066	0.67	0.061	0.696	0.368	0.014*	0.24	0.116	0.131	0.398
30–40 Gy	0.149	0.341	0.166	0.289	0.310	0.043*	0.093	0.554	0.077	0.622
40–50 Gy	0.256	0.097	0.255	0.098	0.243	0.117	0.18	0.249	0.194	0.212
> 50 Gy	0.165	0.488	0.153	0.52	0.084	0.726	0.251	0.286	0.119	0.618

* Statistically significant

## Discussion

The standard treatment for locally advanced cervical cancer is concurrent chemoradiotherapy. A common side effect in patients undergoing chemotherapy is HT. When receiving local radiotherapy alone, metabolic activity increased in the unirradiated bone marrow. The compensatory response is reduced during concurrent chemoradiotherapy owing to the superimposed toxicity, which increases the incidence of HT[12, 13]. The hematopoietic stem cells in the red bone marrow can self-renew and regenerate, which is the basis of maintaining normal hematopoietic function and repairing bone marrow injury. Wang et al. [14]used ex vivo HRMAS 1 H NMRS and assessed microvascular perfusion status and changes occurring in the fat content composition in the bone marrow of rat femurs after total-body X-ray irradiation. Moreover, the bone marrow fat content gradually increased at days 4 and 7 post-irradiation, and the bone marrow microcirculation perfusion was correlated with fat content.

MRI IDEAL IQ FatFrac maps obtained by water-fat imaging can be used to measure the changes of bone marrow fat fraction quantitatively during the treatment course, providing quantitative information on marrow composition. IDEAL IQ is sensitive to marrow composition changes, quantitatively assessing bone marrow damage resulting from chemotherapy and radiation [15]. Carmona et al. [16] used IDEAL IQ to assess the changes in vertebrae bone marrow fat fraction during chemoradiotherapy and detected that the bone marrow mean dose was associated with a 0.43% per Gy increase in PDFF (%). The changes in the latter are associated with peripheral blood cell counts. In this study, PDFF% increased by 58.5% during radiotherapy and had a positive dose–response relationship with dose accumulation. PDFF% is closely related to the changes in blood cell count, which is consistent with the study by Carmona [16].

Increased radiation dose to pelvic bone marrow enhances hematologic toxicity in patients undergoing chemoradiotherapy. The dose constraints of PBM could minimize the incidence of HT to some extent. To implement of individualized bone marrow sparing, the effect of radiotherapy dose on the bone marrow in cervical cancer must be clarified. There are currently no standard criteria for optimal PBM dose limitation regimens. Some retrospective studies and NTCP models have demonstrated that a low dose of PBM was significantly associated with HT events [17, 18]. Kumar et al. [19] detected that G4 HT was related to PBM-V5 > 95% and V20 > 45%. Robinson et al. [13] demonstrated that active bone marrow V20 < 20 Gy was significantly correlated with WBC and ANC nadirs. Zhu et al. [20] concluded that with every 1 Gy increase in mean PBM dose, there was a reduction in ANC and WBC by 9.6/µL and 7.8/µL per week, respectively.

In this study, the analysis of PDFF% changes under different dose gradients demonstrated that the dose of radiotherapy caused a significant increase of 58.5% in PDFF% in the pelvic bone marrow. Moreover, there was a correlation between the V40 of the PBM and WBC nadirs. Significant positive correlations were observed between PDFF (%) changes and ANC nadirs at 5–10 Gy. Moreover, PDFF (%) changes are related to ALC nadirs at 5–40 Gy, which is consistent with previous studies [6, 21, 22]. It may be related to the irradiation range of low-dose area, suggesting that bone marrow sparing in radiotherapy for cervical cancer should reduce the range of low-dose bone marrow irradiation under the premise of ensuring the coverage of the target area.

The PBM dose-volume parameters V40 were found to be related to the ALC nadir, while the PDFF% at 5–40 Gy dose gradients correlated to the ALC nadir. This phenomenon demonstrated that traditional dose-volume indices as astatic indicators have limitations in predicting blood counts changes, whereas PDFF% as a dynamic indicator could reveal more information of BM injury and accurately predict the risk of HT.

From the perspective of radiobiology, bone marrow injury caused by high-dose radiation is more long-lasting and challenging to repair. In this study, WBC, ANC, and PLT nadirs occurred at RT mid-point, which decreased by 64.8%, 68.5%, and 80.1%, respectively; however, they only recovered to 56.4%, 70.2%, and 42.6%, one month after radiotherapy. PDFF% decreased in 6 months after radiotherapy; however, it still increased by 55.95% compared with that at RT-Pre. Nevertheless, the fat content in bone marrow areas receiving high doses (> 30 Gy) continued to increase after radiotherapy, indicating that the bone marrow injury caused by high dose radiation is challenging to reverse or repair in a short time. This study found no correlation between PDFF% in the high-dose area (> 30 Gy) and HT, which was primarily directly related to the small absolute volume of high-dose irradiation. Mazzola et al. [23] demonstrated that volumetric-modulated arc therapy-simultaneous integrated boost appears promising for local control and overall survival in cervical cancer, with an acceptable acute and late HT. Although high dose external beam radiotherapy can achieve a high curative effect, the importance of individualized bone marrow sparing cannot be ignored.

This study demonstrates that MRI IDEAL IQ FatFrac imaging can be used to quantify the changes in bone marrow composition during chemoradiotherapy. We confirmed the correlation between PDFF% and peripheral blood cell reduction, while PDFF% changes and dose accumulation demonstrated a significant dose–response relationship. By measuring of the bone marrow signal values, IDEAL IQ can distinguish red from yellow bone marrow. The combination of MRI and fat quantitative technology could accurately locate the position and range of active bone marrow, thereby providing a visual basis for individualized bone marrow sparing.

This study primarily assessed the changes of PDFF% under different dose gradients over the course of treatment and evaluated the correlation between changes in PBM fat contents and peripheral blood cells. However, the long-term change trend of bone marrow fat content still requires further assessment. Sini et al. [24] detected that the ALC was still at a low level one year after radiotherapy. Therefore, our team anticipates obtaining the fat fraction imaging and peripheral blood cell count of patients long after the end of radiotherapy through further follow-up, establishing a prediction model based on the bone marrow fat content dynamic changes and radiation dose to guide the individualized bone marrow sparing.

In conclusion, the present study used MRI IDEAL IQ fat fraction imaging to evaluate the fat content of bone marrow noninvasively, analyzed the changes of pelvic bone marrow fat content during and after radiotherapy, revealed the dose–effect of bone marrow fat content, and discussed the relationship between bone marrow changes and HT. A strong correlation between low-dose radiation and HT was detected. The individualized sparing of PBM low-dose irradiation during concurrent chemoradiotherapy for cervical cancer should be paid more attention to.

## Data Availability

All data generated or analyzed during this study are available from the corresponding author on reasonable request.

## Abbreviations

PBM: Pelvic bone marrow
IDEAL IQ: Iterative decomposition of water and fat with echo asymmetrical and least squares estimation
PDFF%: Proton density fat fraction
ALC: Lymphocytes
WBC: White blood cells
ANC: Neutrophils
PLT: Platelets
HT: Hematological toxicity
PET/CT: Positron emission tomography
IMRT: Intensity modulated radiotherapy
FIGO: International Federation of Gynecology and Obstetrics
VMAT: Volumetric modulated arc therapy
OARs: Organs at risk
CTV: Clinical target volume
GTV: Gross tumor volume
PTV: Planning target volume
ROI: Region of interest
HGB: Hemoglobin
